# Application and interpretation of functional data analysis techniques to differential scanning calorimetry data from lupus patients

**DOI:** 10.1371/journal.pone.0186232

**Published:** 2017-11-09

**Authors:** Sarah K. Kendrick, Qi Zheng, Nichola C. Garbett, Guy N. Brock

**Affiliations:** 1 University of Louisville, School of Public Health and Information Sciences, Department of Bioinformatics and Biostatistics, Louisville, KY, United States of America; 2 University of Louisville, James Graham Brown Cancer Center, Department of Medicine, Louisville, KY, United States of America; University of Louisville School of Public Health and Information Sciences, UNITED STATES

## Abstract

**Background:**

DSC is used to determine thermally-induced conformational changes of biomolecules within a blood plasma sample. Recent research has indicated that DSC curves (or thermograms) may have different characteristics based on disease status and, thus, may be useful as a monitoring and diagnostic tool for some diseases. Since thermograms are curves measured over a range of temperature values, they are considered functional data. In this paper we apply functional data analysis techniques to analyze differential scanning calorimetry (DSC) data from individuals from the Lupus Family Registry and Repository (LFRR). The aim was to assess the effect of lupus disease status as well as additional covariates on the thermogram profiles, and use FD analysis methods to create models for classifying lupus vs. control patients on the basis of the thermogram curves.

**Methods:**

Thermograms were collected for 300 lupus patients and 300 controls without lupus who were matched with diseased individuals based on sex, race, and age. First, functional regression with a functional response (DSC) and categorical predictor (disease status) was used to determine how thermogram curve structure varied according to disease status and other covariates including sex, race, and year of birth. Next, functional logistic regression with disease status as the response and functional principal component analysis (FPCA) scores as the predictors was used to model the effect of thermogram structure on disease status prediction. The prediction accuracy for patients with Osteoarthritis and Rheumatoid Arthritis but without Lupus was also calculated to determine the ability of the classifier to differentiate between Lupus and other diseases. Data were divided 1000 times into separate 2/3 training and 1/3 test data for evaluation of predictions. Finally, derivatives of thermogram curves were included in the models to determine whether they aided in prediction of disease status.

**Results:**

Functional regression with thermogram as a functional response and disease status as predictor showed a clear separation in thermogram curve structure between cases and controls. The logistic regression model with FPCA scores as the predictors gave the most accurate results with a mean 79.22% correct classification rate with a mean sensitivity = 79.70%, and specificity = 81.48%. The model correctly classified OA and RA patients without Lupus as controls at a rate of 75.92% on average with a mean sensitivity = 79.70% and specificity = 77.6%. Regression models including FPCA scores for derivative curves did not perform as well, nor did regression models including covariates.

**Conclusion:**

Changes in thermograms observed in the disease state likely reflect covalent modifications of plasma proteins or changes in large protein-protein interacting networks resulting in the stabilization of plasma proteins towards thermal denaturation. By relating functional principal components from thermograms to disease status, our Functional Principal Component Analysis model provides results that are more easily interpretable compared to prior studies. Further, the model could also potentially be coupled with other biomarkers to improve diagnostic classification for lupus.

## Introduction

Differential scanning calorimetry (DSC) is used to determine thermally-induced conformational changes of biomolecules within a blood plasma sample. The sample is heated over a controlled temperature range and excess specific heat capacity between the sample and a reference is measured at defined temperature increments. The excess specific heat capacity can be plotted against temperature producing a curve referred to as a thermogram. Recent research has indicated that these curves may have different characteristics based on disease status and, thus, may be useful as a monitoring and diagnostic tool for some diseases [1,2]. One example where DSC thermograms may be helpful in diagnosis and disease monitoring is with lupus patients. Systematic lupus erythematosus, Lupus, is an auto-immune disease in which individuals’ immune systems produce antibodies to cells within the body leading to inflammation. Lupus can affect a wide array of organs/systems within the body and often has symptoms that mimic other diseases. This makes it very difficult to diagnose and monitor Lupus. The American College of Rheumatology provides a list of 11 criteria for potential Lupus diagnosis. An individual is classified as being positive for Lupus if they meet at least 4 of the 11 criteria. This methodology often leads to over-diagnosis, under-diagnosis, and often misses early and mild cases. Therefore, researchers and doctors are looking for new and improved Lupus diagnostic tools [3–5].

Since thermograms are curves measured over a range of temperature values, they are considered functional data and methods developed for functional data analysis (FD analysis) can be applied. In FD analysis, the entire curve or function is considered as one unit of observation instead of multiple observations along a time continuum. Ramsay and Silverman (2005) give an authoritative account of the FD analysis framework and accompanying analysis tools, including how to specify basis systems for building up functions, how to build functional data objects, how to smooth functional curves, how to perform functional principal component analysis, and how to implement linear regression within the functional framework [6]. Ramsay and Silverman’s work focused on models for linear regression when the response variable is either scalar (with functional predictors) or functional. However, other authors ([7,8]) have developed models for functional generalized linear models (FGLM) that are capable of handling categorical response variables with functional predictors.

The use of thermogram profiles as a diagnostic tool is a relatively new research idea that is rapidly gaining interest [2,9–27]. Surprisingly, however, functional data analysis has never been used to analyze thermograms. Fish et al. focused strictly on classification of individuals as cases or controls using a non-parametric method to calculate a similarity metric for classification [11]. However, the methodology of Fish et al. is limited since it does not allow the incorporation of covariates into the classification model. Vega et al. presented a novel method for analyzing thermograms in which they first broke down the thermograms into six individual peaks to represent the curves. They then used parameters corresponding to each peak in a multiparametric comparative method to develop classification criteria [26]. Similar to Fish’s paper, Vega et al. only focused on classification and did not explore the effect of other covariates on their methods. Finally, Garbett and Brock evaluated the use of multiple classification methods designed for high-dimensional data including penalized logistic regression, support vector machines (SVM), and modified linear discriminant analysis for classifying Lupus patients versus controls based on thermogram data [28]. While these approaches allow the incorporation of additional covariates into the model, they treat the excess specific heat capacity at each temperature as a separate covariate rather than analyzing the thermogram curve as a single unit of observation. This leads to potential difficulty in interpreting the resulting solution vector, a limitation that functional data analysis methods are designed to overcome.

In this paper we apply functional regression analysis to thermogram data collected on Lupus and non-Lupus patients. Initially, we treat the thermogram data as a functional response variable and analyze the effect that disease status, along with other covariates (sex, race and year of birth), have on the thermogram profiles. For the second model, we treat disease status as a categorical response variable and use the thermogram profiles as a functional predictor variable. Due to the infinite-dimensional nature of the problem we use functional principal component scores to both reduce the dimensionality and aid in model interpretation. Lastly, we evaluate the derivatives of the thermogram functional data as potential predictors for lupus disease status. Classification accuracy for disease status was evaluated by splitting the data into separate training and test data sets.

The rest of this paper is organized as follows. In Section 2 we introduce the model for each of the regression methods listed above. In Section 3 we apply these models to the Lupus data and present the results. Finally, in Section 4 we will discuss the results, limitations, and future work opportunities.

## Functional regression

In this section we review linear regression models with a functional predictor and scalar/categorical covariate(s). We also explore the functional generalized linear model (FGLM) using the logit link function for a dichotomous response and at least one functional covariate.

### Linear models for functional responses

Linear models with a functional response variable and scalar/categorical covariates is used when a researcher is interested in predicting a functional response based on the values of the covariate(s). Let *y*_*i*_(*t*) be the thermogram value of the *i*th individual at temperature *t* and ***x***_*i*_ = (*x*_*i*1_,…,*x*_*ip*_) be a vector of *p* covariates associated with the *i*th individual. The functional linear model relating the thermogram values to the covariates is then

$$y_{i}(t)=\beta_{0}(t)+\sum_{j=1}^{p}x_{ij}\beta_{j}(t)+\varepsilon_{i}(t),$$ (1)

where the *β*_*j*_(*t*) are functional regression coefficients associated with each covariate and *i* = 1,…,*n* indicates the individual observations. The error term *ε*_*i*_(*t*) is assumed to be a zero mean stationary Gaussian process. For identifiability purposes it is necessary to approximate the high-dimensional covariate functions *β*_*j*_(*t*) using a low-dimensional approximation. One approach is to use a set of basis functions *φ*_1_,…,*φ*_*K*_ to characterize the functional data space. A basis system provides a flexible way to model functional data while still using a parametric framework that allows for inclusion of covariates. The most common basis system is the β-spline basis system, though others are possible. The regression coefficients are then modeled as $\beta_{j}(t)=\sum_{k=1}^{K}b_{j,k}\varphi_{k}$, so that model (1) now becomes

$$y_{i}(t)=\sum_{k=1}^{K}b_{0,k}\varphi_{k}+\sum_{j=1}^{p}\sum_{k=1}^{K}x_{ij}b_{j,k}\varphi_{k}+\varepsilon_{i}(t),$$ (2)

Here, the *b*_*j*,*k*_ are unknown coefficients that relate the basis system and covariate values to the thermogram profiles. The baseline regression coefficient function *β*_0_(*t*) can either represent the overall mean thermogram profile or the thermogram profile of a baseline group, depending on the parameterization for the matrix of covariates [29].

Just as in non-functional regression, in functional regression we may be interested in answering some common statistical questions like,

1. Are the thermograms for cases and controls statistically distinguishable?
2. Are there statistically significant relationships between thermogram profiles and disease status, gender, race, and other covariates?

Functional equivalents of the standard t- and F-tests can be performed to answer such questions. Due to the fact that functional data are inherently high-dimensional, permutation tests are used to determine the critical values for these tests (See Ramsay & Silverman 2009 for details) [29].

### Generalized linear models using functional principal component analysis

In this setting, we use thermogram data as a functional covariate to predict a categorical response, for instance disease status. Here, we focus on the case where the response *Y*_*i*_ is a Bernoulli variable and takes the values of 0 (normal) or 1 (diseased). Since we are now treating the thermogram values as predictor variables, let *x*_*i*_(*t*) be the thermogram value of the *i*th individual at temperature *t* and denote *π*_*i*_ = *P*(*Y*_*i*_ = 1|*x*_*i*_(*t*)) as the probability of the *i*th individual having disease (e.g., lupus) given that individual’s thermogram profile. The logistic regression model is then,

$$\ln\left(\frac{\pi_{i}}{1-\pi_{i}}\right)=\beta_{0}+\int x_{i}(t)\beta_{1}(t)dt.$$ (3)

Given the infinite-dimensional nature of *β*_1_(*t*), this problem is ill-specified in that there are an infinite number of solutions to achieve the same predictions. There are three ways to address this issue; the first two concern a basis coefficient expansion of *β*_1_(*t*) while the third projects the covariate functions into a lower-dimensional space via principal components. Since the latter approach also aids in interpretability, we follow this path. The first step is to identify the functional principal components in the data. This is accomplished by finding the orthogonal loadings or weight functions, ξ, that capture the greatest variation in the data. In other words, we try to find ξ such that the component scores

$$\rho_{\xi }(x_{i})=\int \xi (t)x_{i}(t)dt,$$ (4)

have the largest variation (subject to the constraint that ∫ *ξ*^2^(*t*)*dt* = 1). When the data are *not* functional in nature (i.e., multivariate PCA), these loadings are the solutions to the following eigenequation

$$V\xi_{j}=\mu_{j}\;\xi_{j},$$ (5)

where ***V*** is the covariance matrix of the data with *ξ*_*j*_ being the eigenvectors and *μ*_*j*_ the corresponding eigenvalue solutions. The process is very similar in the functional setting. Here, the eigenfunctions (or harmonics) are calculated as solutions to

$$\int v(s,t)\xi_{j}(t)dt=\mu_{j}\xi_{j}(s),$$ (6)

where *v*(*s*,*t*) is the bivariate covariance function of the functional values *x*_*i*_(*s*) and *x*_*i*_(*t*) (that is, the covariance between two different thermogram measurements at temperature *s* and temperature *t*) [29]. Subsequently, we can characterize each thermogram profile *x*_*i*_ on the basis of these FPCs, and define the principal component scores $\gamma_{ij}=\int \xi_{j}(t)(x_{i}(t)-\bar{x}(t))dt$ as the coefficients which provide the optimal fit to the *x*_*i*_ on this basis.

The function *pca*.*fd* in the *fda* package in R can be used to perform FPCA on a functional object. Therefore, to perform FPCA, the original data must first be converted to a functional data object. This can be accomplished by setting up a saturated basis system (that is, a system where the number of basis functions equals the total number of temperature values, *T*) to represent the data, $x_{i}(t)=\sum_{k=1}^{T}c_{i,k}\varphi_{k}(t)$. Once FPCA has been performed, the eigenvalues μ_*j*_ can be plotted against their indices *j* to create a scree plot. This plot can be used to determine the number of harmonics to use. Once this has been determined, we then regress the outcome variable onto principal component scores *γ*_*ij*_ using a generalized linear model with the logit link function. The model now becomes

$$\ln\left(\frac{\pi_{i}}{1-\pi_{i}}\right)=\beta_{0}+\sum_{j=1}^{J}\gamma_{ij}\beta_{j}$$ (7)

where *J* is the total number of harmonics selected.

### Incorporating functional derivatives

In addition to the thermogram profiles themselves, the derivative curves of the thermogram profiles might be predictive of disease status. We first calculate the first derivative of the curves, then apply a saturated basis expansion just as we did with the original thermogram profiles. Therefore, we define the following

$$x_{i}^{\prime (t)}=\sum_{k=1}^{T}d_{i,k}\varphi_{k}(t),$$ (8)

where the *d*_*i*,*k*_ are the basis coefficients corresponding to the basis functions *φ*_*k*_(*t*) for characterizing the first derivative curves *x*_*i*_′(*t*) [7]. The models in Eqs (3) and (7) can be extended in a natural way to include derivative profiles as well. Specifically, for model (7) we have:

$$\ln\left(\frac{\pi_{i}}{1-\pi_{i}}\right)=\beta_{0}+\sum_{j=1}^{J}\gamma_{ij}\beta_{1j}+\sum_{l=1}^{L}\tau_{il}\beta_{2l}$$ (9)

where *τ*_*il*_ are the principal component scores corresponding to the thermogram first derivative curves for *i* individuals and *L* harmonics. Since normally only the first few principal components are needed to capture the majority of the variation within the data, FGLM using FPCA allows for dimension reduction which decreases the degrees of freedom for error in the model. This decrease can allow for a more stable estimate compared to models without this dimension reduction [7,29].

## Functional data analysis of Lupus thermogram data

### Samples

Data was obtained from the Lupus Family Registry and Repository (LFRR) which was created to gather large quantities of material and data regarding Lupus patients and controls into one place. The hope of the LFRR is that these materials and data will be used to aid in furthering SLE related genetics research. Users must request permission and gain approval to access the data for research purposes. Rasmussen and colleagues recently described the LFRR design and protocols, including protections of human subjects [30]. We used de-identified plasma samples for 600 individuals received from the LFRR [30]. Plasma samples for 300 patients classified as having Lupus using the ACR criteria were obtained. Another 300 plasma samples from controls without lupus who were matched with diseased individuals based on sex, race, and age were also obtained. All samples received were stored at -80°C until analysis by DSC. Use of the LFRR samples and clinical data was reviewed and approved by the University of Louisville Institutional Review Board (IRB# 177.07, 12.0543) in compliance with the Helsinki Agreement.

### Sample preparation for DSC studies

Plasma samples (100 μL) were dialyzed against a standard phosphate buffer (1.7 mM KH_2_PO_4_, 8.3 mM K_2_HPO_4_, 150 mM NaCl, 15 mM sodium citrate, pH 7.5) for 24 hours at 4°C in order to achieve normalization of buffer conditions for all samples. To effectively dialyze such small volumes of plasma we used Slide-A-Lyzer MINI dialysis devices (MWCO 3,500, 0.1 mL; Pierce, Rockford, IL) that were secured in 25-place floats, placed in a 2 L beaker and dialyzed against 1 L of dialysis buffer. Dialysis units were loaded with 100 μL of dialysis buffer and equilibrated overnight at 4°C against 1 L of dialysis buffer. Frozen plasma samples were thawed overnight at 4°C on the evening before dialysis. The next morning, the dialysis units were removed from the beaker, emptied of buffer and loaded with plasma samples. The dialysis units were returned to the beaker containing dialysis buffer and gently stirred to allow motion of the dialysis float and increase the diffusion rate during dialysis. After each dialysis period, the float was removed and placed in a new beaker containing 1L of fresh dialysis buffer. In all, samples were dialyzed against 4 x 1 L of phosphate buffer with buffer changes after three hours of dialysis, four hours of dialysis, another four hours of dialysis and a final overnight dialysis period. Based on cost and reliability we routinely re-assembled washed dialysis units replacing the original dialysis membrane with cut-to-size Snakeskin Pleated Dialysis Tubing (Pierce, Rockford, IL). After dialysis, samples were recovered from dialysis units and filtered to remove particulates using Spin-X centrifuge tube filters (0.45 μm cellulose acetate; Corning Incorporated, Corning, NY). The final dialysis buffer was also filtered (0.2 μm polyethersulfone; Pall Corporation, Ann Arbor, MI) and used for all sample dilutions and as a reference solution for DSC studies.

### DSC analysis

DSC data were collected with a MicroCal VP-Capillary automated DSC instrument (MicroCal, LLC, Northampton, MA, now part of Malvern) which was serviced and calibrated according to the manufacturer’s procedures. Dialyzed plasma samples were diluted 25-fold to obtain a suitable protein concentration for DSC analysis. Plasma samples and matched dialysate to load the instrument sample and reference chambers, respectively, were transferred to 96-well plates and loaded into the instrument autosampler, thermostated at 5°C, until analysis. Sample volumes of 400 μL were loaded into the 96-well plates to provide a sufficient volume for loading of the instrument capillaries and ensure proper filling of the 135 μL thermal sensing area. DSC scans were recorded from 20°C to 110°C at a scan rate of 1°C/min with a pre-scan equilibration period of 15 minutes, mid feedback mode and a filtering period of 2 seconds. The instrument was cycled overnight by running multiple water-water scans (during the overnight dialysis period) followed the next morning by at least three buffer-buffer scans to condition the instrument chambers before running the sample set. In designing our experimental approach for the analysis of blood plasma samples we have carefully examined each aspect of the process: blood sample collection and handling; sample preparation for DSC analysis; instrument settings and analysis replicates; data analysis and interpretation. These studies have recently been published [12]. Importantly, we demonstrated that plasma thermograms were robust to all analytical and pre-analytical variables examined. These studies enabled us to adopt a standard protocol for the analysis of clinical samples. Our standard protocol based on the limited availability of sample aliquots and to provide reasonable analysis throughput involved the collection of duplicate scans for each sample and batching of samples to ensure that DSC analysis is completed within a seven day window after initial thawing of each sample batch. For each sample set we examined buffer scans collected at the beginning and end of a sample set and after single or consecutive samples scans and determined acceptable reproducibility and effective cleaning of the instrument chambers. We also compared sample scans collected after a buffer or sample scan and found it is possible to collect consecutive sample scans after extensive rinsing of the instrument chambers with little effect on thermogram profile.

DSC data were analyzed using Origin 7 (OriginLab Corporation, Northampton, MA). Raw DSC data were corrected for the instrumental baseline by subtraction of a suitable buffer reference scan. Corrected scans were normalized for the total protein concentration to allow direct comparison of samples. Total protein concentration was determined colorimetrically using the bicinchoninic acid (BCA) protein assay kit and microplate procedure from Pierce (Pierce, Rockford, IL), with minor modifications to the incubation time included in the manufacturer’s protocol. Absorbance readings were taken using a Tecan Sunrise microplate absorbance reader (Tecan U.S., Research Triangle Park, NC). Following normalization, plasma DSC scans were corrected for non-zero baselines by application of a linear baseline fit using Origin 7. Choice of an appropriate sample baseline correction is complicated by the presence of a limited region of post-transition baseline followed by aggregation and precipitation events occurring after the thermal denaturation envelope. In developing our analysis procedure for plasma samples we have evaluated all available baseline correction options within the analysis software and found the linear baseline option to give the most consistent results when tested on repeated measurements, different samples and by independent user determinations. We accept that our approach might have limitations but have selected the most consistent baseline correction method that can be applied across all of our studies. The full DSC dataset has been included as supplementary information (S1 Data). This dataset includes a subject.ID variable (modified to maintain patient anonymity), a status variable (case/control), a temperature variable and a DSC variable for each individual. Approved users are able to request access to additional data–i.e. the clinical data–from the LFRR to pursue research related to SLE.

There were a total of 8 samples that were flagged as having poor quality scans and removed from the data set leaving data for 592 patients. Of the 592 individuals, 298 were cases and 294 were controls. The final thermograms were truncated to the temperature range of interest from 45°C to 90°C and interpolated into regular 0.1°C increments. This yields 451 total temperature values.

### Functional linear model with thermogram data as the response and disease status as the predictor

In the Lupus thermogram framework, the response variable of interest is thermogram shape and structure predicted by disease status (case or control). Therefore, in model 1, i = 1, 2,…, 592, j = 1, 2 for disease status indicator, t = temperature, K = 35 (determined using Generalized Cross Validation (GCV)), and the values of x_ij_ are 0 or 1 indicating either control or case, respectively. Our design matrix is then a 592 by 3 matrix with the first column being all 1’s, the second column contains ones for cases, -1’s for controls, and the third column contains 1’s for controls and -1’s for cases.

**Fig 1:**
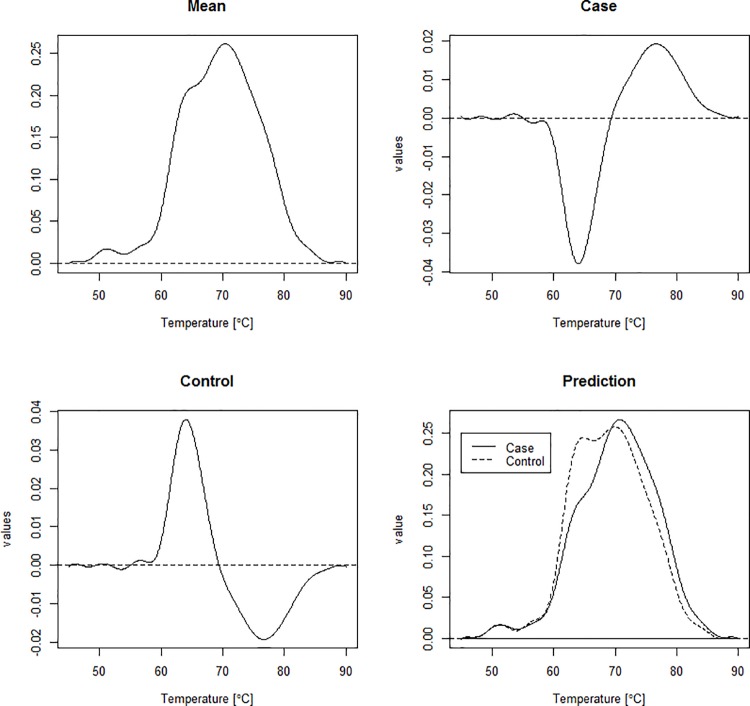
Smoothed regression coefficients estimated for predicting thermograms from disease status. The first panel is the intercept coefficient, corresponding to the overall mean thermogram. The second and third panels show the estimated perturbation (regression coefficients) of the overall mean needed to fit a curve for cases and controls respectively. The last panel shows the predicted mean thermograms for cases and controls.

Since we used 35 B-spline bases, we have 35 terms for each of the three coefficients–intercept, cases, and controls. These beta values can then be plotted against temperature (a sequence that ranges from 45 to 90°C). Since there are only a few values for each coefficient, the plots will look very rough. Therefore, we implement some smoothing to yield more interpretable plots. These plots give the mean thermogram (intercept), and the perturbations of the overall mean required to fit a curve for cases and a curve for controls. We can also use the predicted response values, returned to us from the regression, to get the predicted curves for both cases and controls (**Fig 1**).

**Fig 2:**
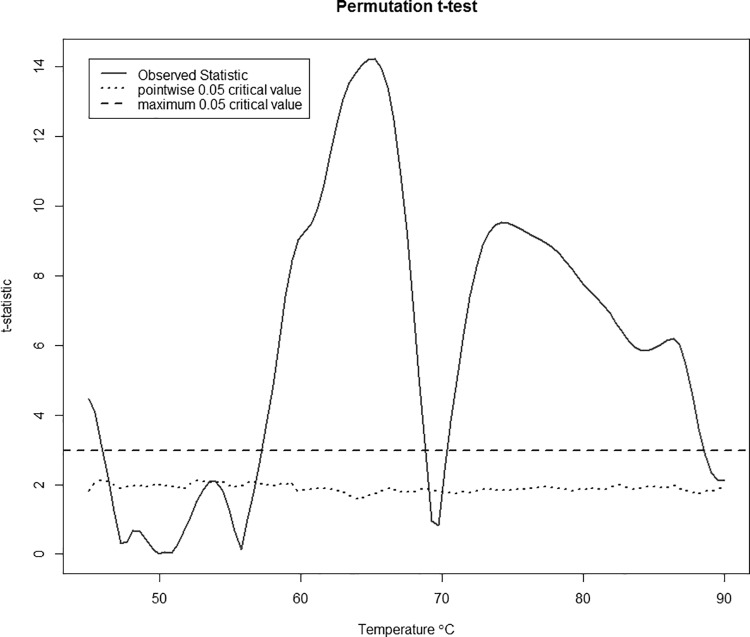
A test for the difference in thermogram profiles between cases and controls. The red line is the observed test statistic at a given temperature, while the dotted blue line is the pointwise critical value determined using 200 permutations. A test for overall differences in the curves between cases and controls can be done by comparing the maximum of the observed statistics (here, 14.22 at a temperature of 65.2°C) with the corresponding critical value for this maximum (dashed blue line).

The functional equivalent to the t-test is plotted in **Fig 2**, which essentially gives a t-test at each temperature along the curve. From the plot we see that the most significant differences between the curve for cases and the curve for controls lies in the ~[60, 69°C and ~[72, 85°C ranges. **Fig 2**also shows the maximum value of the test statistic (highest value of the red line), the critical value for each individual t-test performed (dotted blue line), and the overall critical value based on the maximum of the test statistic (dashed blue line, determined using 200 permutations). Since the maximum value of the test statistic was 14.22, and the critical value from the permutation test was 2.92, this indicates a significant overall difference between the curves for cases and the curves for controls.

#### Inclusion of additional covariates

**Fig 3:**
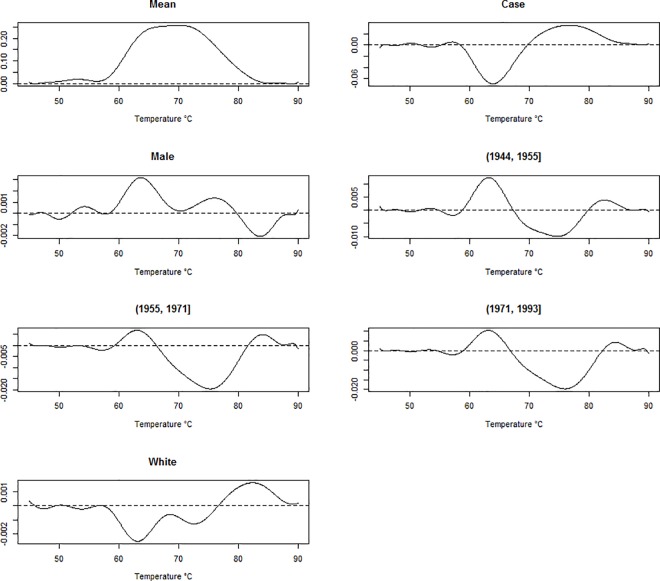
Smoothed regression coefficients estimated for predicting thermograms from disease status, sex, race, and year of birth. From left to right, top to bottom, panel 1 is the intercept coefficient, corresponding to the overall mean thermogram. The second panel shows the estimated perturbation (regression coefficients) of the overall mean needed to fit a curve for cases. The third panel shows the estimated perturbation of the overall mean needed to fit a curve for females. Panels 4–6 show the estimated perturbation of the overall mean thermogram needed to fit a curve for individuals with a birth year in (1944, 1995], (1955, 1971], and (1971, 1993], respectively. Finally, panel 7 shows the estimated perturbation of the overall mean thermogram needed to fit a curve for individuals identifying as White.

Now, we extend the above model to include additional covariates. In our application we chose to include sex, race, and year of birth as covariates in the model. With the addition of covariates, we use a slightly different parameterization than in the reduced model. Now, disease status (case or control), will require one coefficient, sex (male or female) will require one additional coefficient, race (Black or White) will require one additional coefficient, and year of birth (1924–1944, 1945–1955, 1956–1971, or 1972–1993) will require three additional coefficients, making p = 7 in model 2. For each covariate, one level is considered the baseline group and the coefficients for the other levels contrast the corresponding group with the baseline. These contrasts are plotted in **Fig 3**for each of the covariates.

**Fig 4:**
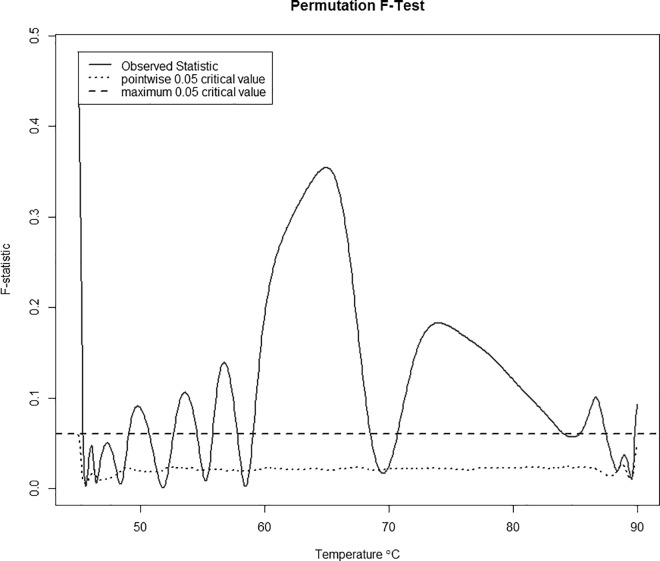
Permutation test for a predictive relationship between disease status, sex, race, and year of birth and thermogram structure. The red line is the observed test statistic at a given temperature, while the dotted blue line is the pointwise critical value determined using 200 permutations. A test for overall differences in the curves between cases and controls can be done by comparing the maximum of the observed statistics (here, 0.35 at a temperature of 64.9°C) with the corresponding critical value for this maximum (dashed blue line).

With more than two groups in the model we can no longer perform the functional t-test but can, instead, implement the functional F-test. Just as with the functional t-test, we can get a plot for these values at each temperature (**Fig 4**) and calculate the F-statistic = max(F(t)). Also, we again use a permutation test to determine the critical value to perform the hypothesis test. **Fig 4**indicates that the strongest predictive relationship between the covariates and thermogram structure lies within the [60, 85°C range. The test yields a maximum observed statistic of 0.35 with a corresponding critical value = 0.06 indicating a strong predictive relationship between the covariates and the response variable. A limitation of this model is that significance of individual covariates cannot be tested.

The results of linear regression with functional response variable and scalar/categorical covariate(s) shows a significant difference between curves for cases and curves for controls as well as a strong predictive relationship between disease status and covariates on thermogram structure. Both models indicate that the largest differences and strongest relationships occur between 60°C and 85°C. Therefore, we chose to only include DSC data within the [60°C, 85°C] temperature range when running the rest of the regression models.

### Generalized linear models using thermogram data as the predictor and disease status as response

Now we shift the focus to the case where the response variable of interest is disease status (1 for lupus, 0 for control) and the thermogram functional data is the predictor. We used the thermogram functional principal component scores as the predictor variable to reduce the dimensionality of the problem and aid in interpretation, with the overall goal to investigate how well thermogram shape and structure predict disease status. To evaluate the predictability of the model we split the data into a 2/3 training set and 1/3 test set giving us 200 cases / 200 controls in the training set and 98 cases / 94 controls in the test set. We re-ran the regression using only the training set and used the results to predict the response values for the test set. An observation within the test set was classified as a case if their predicted value was greater than 0.50; classified as a control if their predicted response value was less than 0.50. We repeated this 1000 times using a different split each time and took the mean of percent correct classification to get the final predicted classification accuracy. The confidence interval for prediction accuracy was calculated using the sample of 1000 classification percentages and the standard formula for a confidence interval $CI=\bar{x}\pm \left(1.96*\frac{stdev}{\sqrt{n}}\right)$. We also looked specifically at the set of patients that had rheumatoid arthritis and/or osteoarthritis but did not have lupus (17 total patients) to determine how well our classifier did at correctly classifying these patients as controls.

**Fig 5:**
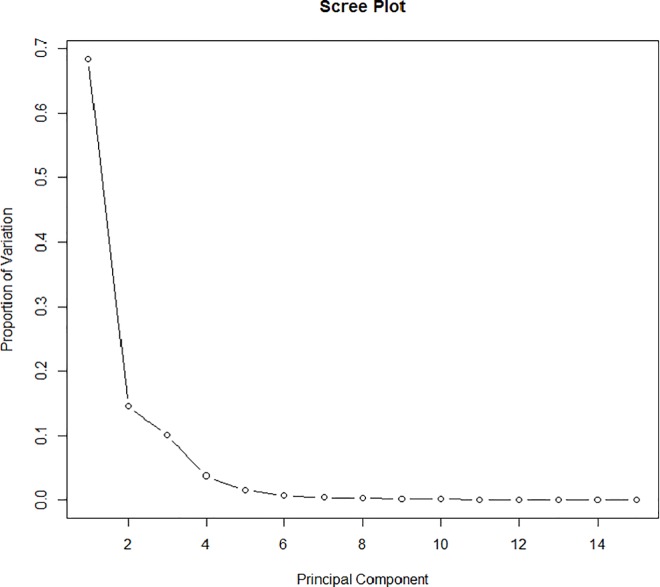
Scree plot of the first 15 PC’s resulting from FPCA on the thermogram data of 298 lupus cases and 294 controls.

**Fig 6:**
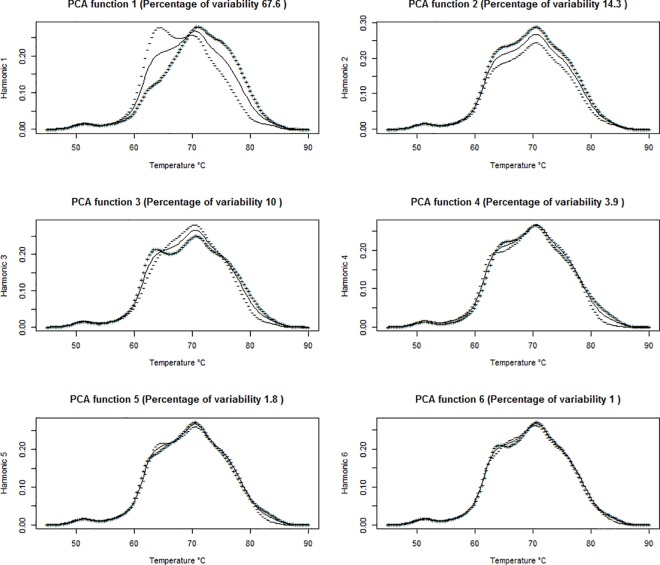
The first 6 functional PC’s and the percent variation they capture. The solid line in each curve represents the mean thermogram curve, while the curves labeled with a ‘+’ or a ‘-‘ indicate what happens when one standard deviation of the harmonic is added (+) or subtracted (-) from the mean.

We first performed FPCA on the thermogram data. **Fig 5**shows the scree plot of the first 15 principal components (PC’s). From the scree plot, we concluded that only the first six PC’s were needed since together they explained 99% of the variation in the data. **Fig 6**plots the overall mean thermogram curve as well as two additional curves for each PC. These two additional curves show what happens to the mean curve when one standard deviation of the armonic is added (+) or subtracted (-). We see that the first harmonic captured 64.6% of the total variation about the mean and shows the contrast between cases and controls. The second harmonic, explaining an additional 14.3% of variation, indicated a vertical shift in the mean. The third harmonic captured the vertical shifts about the two main peaks, and the remaining harmonics captured much smaller noise and variation.

Now that we determined the number of PC’s to include, we used the function *glm* in the stats package in R to fit the model in Section 2.2 on each of the 1000 different training and test sets. **Table 1**gives the estimates, standard errors, odds ratio (95% confidence interval), and p-values for the coefficients from one run. Finally, for each split, individuals are classified as case or control if their predicted response value is more than 0.50 or less than 0.50, respectively. Comparing these predicted classifications to true disease status and taking the mean, we get 79.22% correct classification from the model. When looking just at patients that had RA and/or OA but did not have lupus, the model correctly classified these patients as non-Lupus 75.92% of the time. This is comparable to the overall prediction accuracy indicating that our classifier is specific to Lupus.

**Table 1:** Estimated regression coefficients for the first 6 principal components in the FGLM model using FPC scores.

Coefficient	Estimate	Std. Error	OR (95% CI)	p-value
Intercept	0.07	0.10	1.07 (0.88, 1.31)	0.50
PC 1	1.27	0.12	3.56 (2.82, 4.49)	<0.001
PC 2	-0.40	0.10	0.67 (0.55, 0.82)	<0.001
PC 3	-0.09	0.11	0.91 (0.74, 1.12)	0.387
PC 4	0.04	0.10	1.04 (0.85, 1.28)	0.678
PC 5	0.57	0.11	1.77 (1.43, 2.17)	<0.001
PC 6	-0.35	0.10	0.70 (0.57, 0.86)	0.001

**Fig 7:**
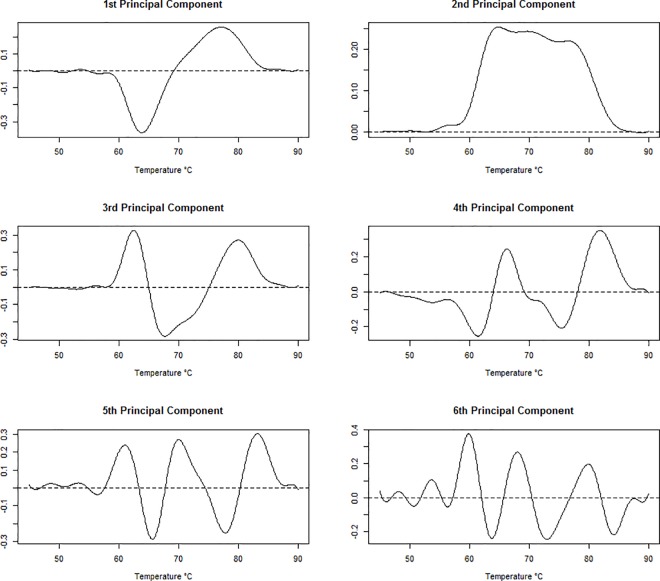
The first 6 principal component curves for thermogram profiles.

**Fig 7**shows the coefficient vectors (or loadings) for the first six principal components. The first principal component curve models individuals starting with excess specific heat values around average that then drop below average starting around 60°C, and then increase to above average values starting around 70°C before eventually decreasing back to average. Individuals with thermograms matching this pattern will have a large first PC score and individuals experiencing the opposite of this will have small first PC score. This curve very closely resembles the regression curve for cases in **Fig 1**and **Fig 3**, therefore we can conclude that cases will tend to have large first PC scores and controls will likely have small first PC scores. From **Table 1**, we see that the first principal component is highly significant for disease status and that individuals with curves described as above have an odds ratio of exp(1.269) = 3.56. This indicates that the odds of being classified as a case is 3.56 times greater for each unit increase in standard deviation. The second principal component was also found to be significant and represents individuals that have above average excess specific heat values between 55°C and 85°C. Individuals experiencing this type of vertical shift from the average curve have an odds ratio of exp(-0.40) = 0.67 of being a case relative to an individual with a thermogram structure more similar to the overall mean thermogram in this data set.

**Fig 8:**
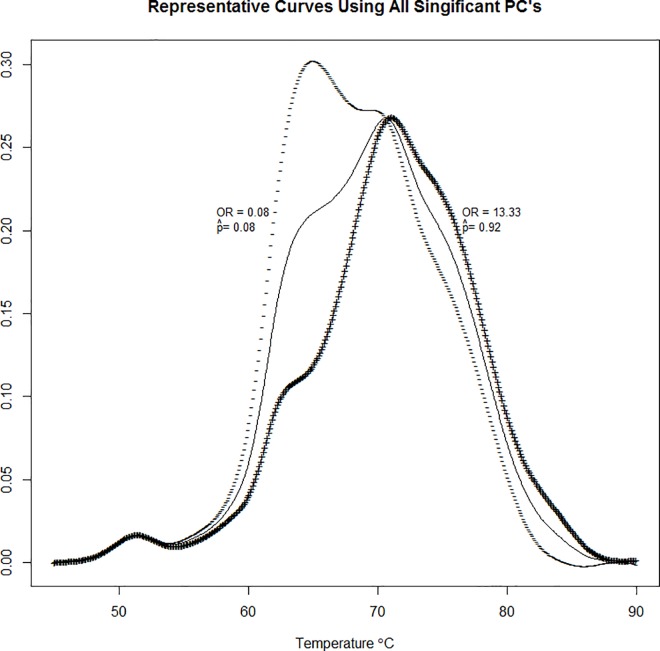
Curves representative of cases and controls determined by adding (+) or subtracting (-) one standard deviation of the significant harmonics determined by FGLM using FPCA regression.

The third principal component was not significant for disease status and the remaining three principal components explain only a small portion of the total variance (6.7%). However, principal components 5 and 6 were found to be highly significant. Using just the statistically significant principal components 1, 2, 5, and 6 we can produce curves representative of cases and controls by either adding (PCs 1 and 5 for cases, PCs 2 and 6 for controls) or subtracting (PCs 2 and 6 for cases, PCs 1 and 5 for controls) one standard deviation of the significant harmonics to the mean thermogram (**Fig 8**). **Fig 8**indicates that an individual with a curve similar to the curve represented by (+) has a 92% probability (conditional on being from the given data set) of being classified as Lupus, while an individual with a curve similar to that represented by (-) has an 8% probability (conditional on being from the given data set) of being classified as Lupus.

**Fig 9:**
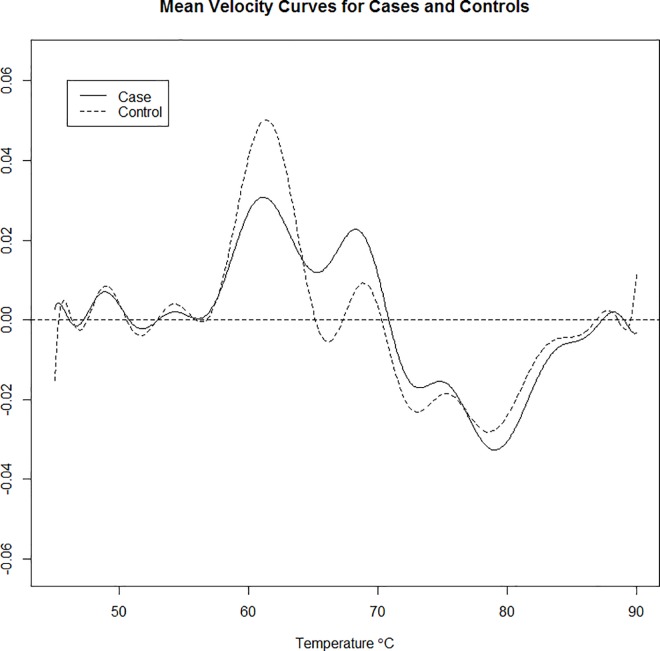
Mean velocity (first derivative) curves for cases and controls.

**Fig 10:**
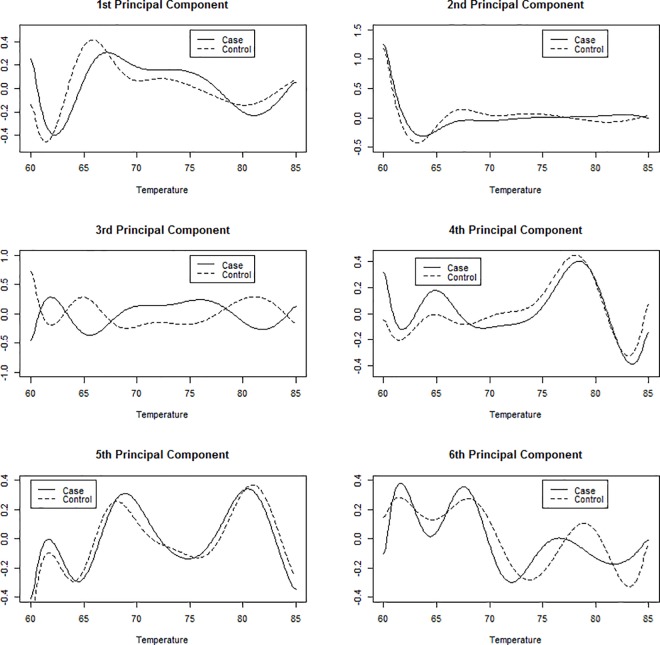
First six harmonics of the velocity curves for cases and controls.

Finally, we include the FPCA scores for derivative curves into the model to explore their effect on regression and prediction accuracy. The mean first derivative curve, along with the first six harmonics of the first derivative curve (stratified by disease status) are shown in **Fig 9**and **Fig 10**, respectively. Just as before, the data was split into 1000 training and test sets using a 2/3 vs. 1/3 split. We report results on three different results comparing the original thermogram curves, the first derivative curves, and the model including both sets of curves (**Table 2)**. From the results, it is evident that incorporation of the derivative curves into the model is not beneficial.

**Table 2:** Prediction accuracy for models including FPCA scores of derivative curves as predictor variable(s). Numbers are mean values from 1000 test data sets along with 95% CIs for the mean.

Variable(s) Included in the Model	Prediction Accuracy	Sensitivity	Specificity
Thermogram RA and OA Controls (N = 17)	79.22% (78.38, 80.06%) 75.92% (74.71, 77.13)	79.70% (79.18, 80.22) 79.70% (79.18, 80.22)	81.48% (80.42, 82.53) 77.67% (76.55, 78.78)
Velocity RA and OA Controls (N = 17)	76.28% (76.12, 76.44)% 76.60% (75.80, 77.40)%	72.58% (72.32, 72.84) 72.58% (72.32, 72.84)	80.06% (79.80, 80.32) 76.69% (75.91, 77.46)
Thermogram and Velocity RA and OA Controls (N = 17)	75.78% (75.62, 75.95)% 78.58% (77.87, 79.28)%	74.04% (73.78, 74.31) 74.04% (73.78, 74.31)	77.51% (77.24, 77.77) 78.34% (77.68, 79.01)

## Discussion

Linear regression with a functional response variable showed that there is a clear separation between the predicted curve for cases and the predicted curve for controls. The curve for controls is clearly bimodal whereas the curve for cases is more unimodal. Also, the curve for controls is shifted slightly left with respect to the curve for cases, having an overlap only in the tails and around 70°C. While these differences can be seen through median profile plots and other exploratory data analysis, functional linear regression allows us to test the effect of these differences. The functional t-test indicated that the overall difference between the plots for cases and controls is significant, with the most significant differences occurring between 60°C and 85°C. Functional linear regression with thermogram profiles as the response variable also allows exploration of the effect of additional covariates on the thermogram profiles. The maximum absolute value of the regression coefficient for the sex covariate is ≈ 0.004 which is very small. This indicates that any effect sex may have on the thermogram profile is very minimal. We see similar results for the race coefficient. Since individuals were matched based on sex and race, these results make sense. The maximum absolute value for the beta values representing the year of birth coefficient is slightly larger, ≈ 0.02 which occurs for all year of birth categories around 75°C. However, this still does not appear to have a noticeable effect. With the addition of covariates, we ran a functional F-test instead of the functional t-test, but the results were very similar. In other words, the effect on the thermogram curves due to disease status was roughly 3 times greater than the year of birth cohort and over an order of magnitude greater than sex and race. Again we saw the most significant differences between 60°C and 85°C. Due to this fact, we chose to restrict our temperature values to only those falling in this range when moving forward with the rest of the analyses.

Fitting a functional generalized linear model to this data revealed that thermogram shape can help predict disease status. Note that the covariates sex, race, and year of birth were not included in this model because the case/control samples were matched on these covariates. The FGLM that regressed disease status onto the functional principal component scores for the original thermogram data gave the highest correct classification percentage of all the models. This is likely due to the dimension reduction allowed by first performing FPCA on the thermogram data. The FGLM that regressed disease status onto the functional principal component scores for the velocity curves (1^st^ derivative of the thermogram profiles) and the FGLM that regressed disease status onto the functional principal component scores for the velocity curve combined with the scores for the original thermogram both performed worse than the model including functional principal component scores for the original thermogram alone.

The classification accuracy achieved by the GLM model using the functional PCAs was comparable to that achieved previously by Fish et al. [11], but less than that achieved by Garbett and Brock [28]. However, the models fitted by Garbett and Brock produced difficult to interpret solution vectors, with many oscillations (c.f. **Fig 7**in that paper). In contrast, the functional principal component curves (**Fig 7**) are easier to interpret and can be coupled together to form a ‘composite’ curve with a corresponding odds ratio for disease (**Fig 8**). This gain in interpretability can point to target areas of the thermogram curves which can be explored further for biochemical constituents which drive the compositional changes in the curves. Lastly, the predictions obtained from this case / control data set cannot be directly extended to the general population. However, the sensitivity / specificity of the model (using a pre-determined cut-point for disease determination) could be paired with disease prevalence for a given demographic strata to calculate positive and negative predictive values using Bayes’ theorem. Further, the disease odds based on thermogram data could potentially be coupled with other, independent information on disease status (e.g., based on clinical or immunological markers) to obtain posterior odds of disease [12]. Though, independence of the thermograms from existing diagnostic criteria would have to be tested and perhaps cannot be fully verified.

In this paper, we explored logistic regression as a method to formulate predictions. Future work includes investigating other methods for classification within the functional data framework, such as linear discriminant analysis [31] and support vector machines [32]. There is also potential for developing new methodology for classification within the FD analysis framework that may perform better than existing methods–for instance functional decision trees. In addition, uture work investigates extension of longitudinal/repeated measures regression into the FD analysis framework.

Although the focus of this paper was to apply new analysis techniques for the diagnostic classification of thermograms, it is important to address the nature of the thermogram changes and how these might relate to the disease state. Although the mechanism is currently unknown we believe that the overall stabilization in thermogram profile observed in the disease state reflects biomarker modifications within the plasma proteome resulting in the stabilization of plasma proteins towards thermal denaturation. The nature of these modifications has not been determined but the large changes in plasma thermograms argues against the involvement of weak, non-covalent interactions of small molecule biomarkers with plasma proteins and is more likely to reflect covalent modifications of plasma proteins or changes in large protein-protein interacting networks. While it is important, and of great interest, to understand the origin of disease thermogram changes it is not essential for the practical use of thermograms, as long as there are consistent and reproducible signatures that can be used as a diagnostic indicator for a particular disease.

In conclusion, the logistic regression model with FPCA scores performed best. Although the prediction accuracy from this model was not as high as the prediction accuracy from models in other papers, our model yields results that are more easily interpretable. These methods give more insight into the power of thermograms as a diagnostic tool and could be used to in conjunction with the already existing diagnostic method for Lupus to increase sensitivity and specificity.

## Supporting information

S1 Data The full DSC dataset with case/control status variable. (CSV)
